# Catch–up growth in the first two years of life in Extremely Low Birth Weight (ELBW) infants is associated with lower body fat in young adolescence

**DOI:** 10.1371/journal.pone.0173349

**Published:** 2017-03-09

**Authors:** Anke Raaijmakers, Lotte Jacobs, Maissa Rayyan, Theun Pieter van Tienoven, Els Ortibus, Elena Levtchenko, Jan A. Staessen, Karel Allegaert

**Affiliations:** 1 Department of Pediatrics and Neonatology, University Hospitals Leuven, Leuven, Belgium; 2 KU Leuven Department of Development and Regeneration, University of Leuven, Leuven, Belgium; 3 Research Unit Hypertension and Cardiovascular Epidemiology, KU Leuven Department of Cardiovascular Sciences, University of Leuven, Leuven, Belgium; 4 Department of Sociology, Vrije Universiteit Brussel, Brussels, Belgium; 5 R&D Group VitaK, Maastricht University, Maastricht, The Netherlands; 6 Intensive Care and Department of Pediatric Surgery, Erasmus Medical Center Sophia Children’s Hospital, Rotterdam, The Netherlands; TNO, NETHERLANDS

## Abstract

**Aim:**

To investigate growth patterns and anthropometrics in former extremely low birth weight (ELBW, <1000 g) children and link these outcomes to neurocognition and body composition in childhood.

**Methods:**

ELBW children were examined at birth (n = 140), at 9 and 24 months (n≥96) and at approximately 11 years within the framework of the PREMATCH (PREMATurity as predictor children’s of Cardiovascular and renal Health) case–control (n = 93–87) study. Regional growth charts were used to convert anthropometrics into Z–scores. Catch–up growth in the first two years of life was qualified as present if ΔZ–score >0.67 SDS. At 11 years, anthropometrics, neurocognitive performance, body composition, grip strength and puberty scores were assessed.

**Results:**

ELBW neonates displayed extra–uterine growth restriction with mean Z–scores for height, weight and head circumference of –0.77, –0.93 and –0.46 at birth, –1.61, –1.67 and –0.72 at 9 months, –1.22, –1.61 and –0.84 at 24 months, and –0.42, –0.49 and –1.09 at 11 years. ELBW children performed consistently worse on neurocognitive testing with an average intelligence quotient equivalent at 11 years of 92.5 (SD 13.1). Catch–up growth was not associated with neurocognitive performance. Compared to controls, ELBW cases had lower grip strength (13.6 vs. 15.9 kg) and percentage lean body weight (75.1 vs. 80.5%), but higher body fat (24.6 vs. 19.2%) and advanced puberty scores at 11 years (all *P*≤0.025). Catch–up growth for weight and height in the first two years of life in cases was associated with a lower percentage body fat compared to cases without catch–up growth (16.8% catch-up growth for weight *vs*. 25.7%, *P*<0.001; 20.9% catch-up for height *vs*. 25.8%, *P* = 0.049).

**Conclusions:**

In young adolescence, former ELBW children still have difficulties to reach their target height. Compared to normal birth weight controls, ELBW adolescents show lower neurocognitive performance and grip strength and a higher percentage body fat, a potential risk factor for adverse health outcomes in adulthood. Our key finding is that catch–up growth in ELBW children in the first two years of life is associated with a lower percentage body fat and is therefore likely to be beneficial.

## Introduction

The Global Burden of Disease Study [1] revealed that cardiovascular disease in adulthood is attributable to modifiable risk factors such as hypertension [2], body mass index [3] or adverse lipid metabolism [4]. Preterm birth and low birth weight are also such cardiovascular risk factors [5]. Prevention of preterm birth and its consequences (e.g. hypertension, hyperlipidemia) has potentially large impact on cardiovascular health later in life. The repercussions of slow or rapid (catch–up) growth and its timing in neonatal life and throughout childhood are only partly unveiled [6]. Large epidemiological studies concerning the optimal (catch–up) growth patterns (reviewed in [7, 8]) are currently lacking. Extremely low birth weight (ELBW, <1000 g) preterm infants are either small for gestational age (SGA, birth weight <–2 standard deviation scores for gestational age) or ‘intra–uterine growth restricted’, or are appropriate for gestational age (AGA). Irrespective whether SGA or AGA at birth, they remain at high risk of ‘extra–uterine growth restriction’ a potential forerunner of additional morbidities later in life [9].

Since catch–up growth and fat mass accretion in preterm born children do not occur simultaneously [10, 11], body composition should hereby be considered as well. Even late preterm infants, whether SGA or AGA, are characterized by predominant fat mass accretion compared to term neonates [12]. A mismatch in catch–up growth and fat mass accretion in former preterms may lead to metabolic dysregulation in adulthood [13, 14]. Our aim was to investigate growth patterns, catch–up growth and anthropometrics in former ELBW children and to link these outcomes to neurocognition and body composition in childhood. The study cohort consisted of ELBW infants (born 2000–2005), parenterally fed with a relatively low protein diet (2.8 g/kg/day) [15] in comparison to contemporary guidelines (3.5–4.5 g/kg/day) [16]. Their characteristics were compared to a control group born at term of equal sex and age.

## Methods

### Ethics

The study was conducted in accordance with the Helsinki declaration for investigations in human subjects. The local Ethics Committee of the University Hospitals Leuven (Belgium) approved the study protocol (June 2014, S56577). Based on good clinical practice guidance and Belgian legislation, parents or custodians provided written informed consent, whereas children gave informed assent. The study was registered at ClinicalTrials.gov (NCT02147457).

### Cohort description

**Fig 1:**
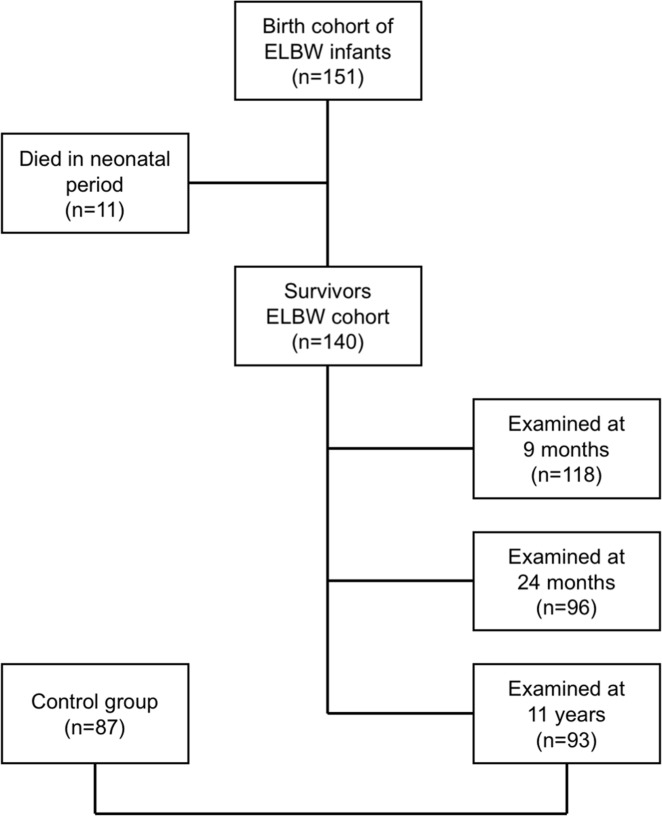
Flowchart of the Extremely Low Birth Weight (ELBW) cohort.

A cohort of former ELBW children (n = 140, gestational age ranging from 23 to 33 weeks) was examined at birth, at the (corrected) ages of 9 and 24 months [17]. Until the age of two, we applied age correction for the extent of prematurity for all measurements (chronological age minus weeks of prematurity equals corrected age). We included 118 children at 9 months of age and 96 at 24 months of age (Fig 1). The PREMATurity as predictor of children’s Cardiovascular and renal Health (PREMATCH) case–control study [18] included 93 of these former ELBW children and 87 controls (on average aged 11 years) of equal sex and age. Both the ELBW cohort and a control group were thoroughly investigated [18]. The control group was recruited through the index child (neighbor, friend) or in a primary school close to the research center [18]. We, therefore, could not perform paired analysis. Nonetheless, the children were of equal sex and age (Table 1). Fig 1 shows a flowchart of the ELBW cohort over time.

**Table 1:** Characteristics in former ELBW children and controls.

Characteristic	ELBW children (n = 93)	Controls (n = 87)	*P* ^a^
**Sex and age**			
Female, n (%)	44 (47.3)	44 (50.6)	0.66
Mean age (±SD), years	11.3±1.4	10.9±1.3	0.025^c^
**Height and head circumference, mean±SD**			
Height, cm	145.1±9.3	149.2±10.1	0.027 ^c^
Z–score of height	–0.45±0.96	0.56±1.05	<0.001^b^
Head circumference, cm	51.7±1.8	53.4±1.6	<0.001^b^
Z–score of head circumference	–1.12±1.03	0.06±0.89	<0.001^b^
**Weight and related parameters, mean±SD**			
Weight, kg	36.7±9.6	39.9±9.3	0.012^c^
Z–score of weight	–0.52±1.05	0.34±0.87	<0.001^b^
Body mass index, kg/m^2^	17.0±2.8	17.7±2.5	0.044^c^
Z–score of body mass index	*–0*.*40*±*1*.*05*	*0*.*04*±*0*.*93*	*0*.*004*^*b*^
Skinfolds			
• Triceps, cm	1.07±0.43	1.20±0.40	0.009^c^
• Subscapular, cm	0.77±0.33	0.78±0.30	0.40^c^
• Supra–iliac, cm	0.76±0.42	0.78±0.40	0.81^c^
Waist circumference, cm	64.8±7.7	66.2±7.4	0.23^c^
Hip circumference, cm	73.7±8.7	76.9±8.5	0.010^c^
Neck circumference, cm	28.7±2.0	29.1±2.1	0.39^c^
**Body composition, mean±SD**			
Percent lean body weight	75.1±10.2	80.5±8.9	**0.001**^c^
Percent fat body weight	24.6±9.8	19.2±9.1	**0.001**^c^
Percent total body water	72.7±9.7	70.3±8.6	0.10^b^
Perfect intracellular water	37.1±6.5	36.1±5.9	0.29^c^
**Grip strength (kg), mean±SD**			
• Right	13.9±4.4	16.0±4.1	**<0.001**^c^
• Left	13.2±4.0	15.8±4.4	**<0.001**^c^
**Puberty scores** ^**d**^**, mean±SD**			
• Breast/genital	2.3±0.87	2.1±0.79	**0.025**^c^
• Pubic	2.2±0.86	1.9±0.78	**0.025**^c^

Extremely Low Birth Weight, ELBW, kcal, kilocalories

^a^*P* values are given for the comparison between ELBW cases and controls

(^b^T test or ^c^Mann–Whitney–U test for continuous variables and Pearson Chi square test for categorical variables)

^d^corrected for age.

### Neurocognitive performance

Trained investigators assessed the cognitive and motor capabilities of the ELBW cohort using the Bayley Scales of Infant Development, Dutch version (BSID–II–NL, [19]) at the corrected age of 24 months. We investigated neurocognitive performance at 11 years (cases and controls) by the Wechsler Non–Verbal test, Dutch version (Pearson, The Netherlands). Matrix reasoning and spatial span was assessed to estimate the intelligence quotient (IQ) equivalent (i.e. total score, [20]). Parental education was assessed by a standardized questionnaire (i.e. low, middle or high) [18].

### Growth, target height and catch–up growth

Body weight and height of cases, controls and their biological parents were recorded [18]. Growth charts of Flanders [21] were used to convert height, weight and head circumference into Z–scores. Target height (Z–score) was calculated according to the corrected mid parental height ([paternal height (–13 centimeters in girls) + maternal height (+13 centimeters in boys)]/2) to estimate the genetic potential of growth [22] and was converted to Z–score as well. Changes in Z–scores (ΔZ) during follow–up were calculated and the presence or absence of catch–up was subsequently classified during the first two years of life. Catch–up growth was qualified as present if the ΔZ–score during the first two years of life was >0.67 SDS [23].

### Body composition, muscle strength and puberty scores

Trained staff assessed body composition using the Bodystat QuadScan 4000 (Bodystat Ltd, Isle of Man, IM99 1DQ, British Isles). This device applies scientifically validated principles of bioelectrical impedance [24, 25]. Skinfolds (Harpenden Skinfold Caliper, Bedfordshire, UK) measurements were read after 3 seconds at three places (at triceps, subscapular and supra–iliac skinfolds). The Jamar Hydraulic Hand Dynamometer (Sammons Preston, Chicago, USA) was used to measure grip strength according to the owner’s manual. A single pediatrician (A.R.) assessed puberty according to the Tanner scores [26, 27]. Additional details can be found in the published study protocol [18].

### Statistics

Continuous variables were expressed as mean (standard deviation, SD) and were tested for normal distribution. Group means between cases and controls were assessed using appropriate (non–)parametric tests (T–tests or Mann–Whitney–U test). Outlier limits were set by extending the 25^th^ and 75^th^ percentile with *k* times the interquartile range [28], with *k* = 2.2 [29] for covariates. Outlier limits for Z-scores were set at Z = |3.29|. Categorical variables were expressed as proportions and differences in frequency distributions were assessed using Pearson Chi–Square tests. Pearson’s’ product–moment correlation coefficients (*r*) were calculated between non–outlier Z–scores and neurocognitive scores at 9 months and 24 months and IQ equivalent scores at 11 years for cases only. A subgroup analysis by sex was done. A linear mixed model analysis with individuals modeled as random and covariates modeled as fixed effects was performed to compare mean Z–scores over time (birth, 9 months, 24 months and 11 years) for cases only. Post–hoc tests were used for pairwise comparisons of mean Z–scores between time points against α = 0.05 using Bonferroni correction for multiple comparisons.

Catch-up growth in weight, height, and head circumference was defined as a positive change in Z-score of 0.67 between time points [23] and converted into a dichotomous variable (yes/no catch-up growth). The compared time points included birth and 9 months, birth and 24 months, and 2 years and 11 years of age. The association of catch-up growth with percentage body fat at the age of 11 years is investigated by T-tests with correction of degrees of freedom in case of violation of homoscedasticity. The percentage body fat is corrected for weight, height, age, sex, and an interaction term of weight × height by saving the predicted values of a multivariate linear regression model (F = 42.613, df = 5, *P*<0.001, adjusted R^2^ = 0.58). Inclusion of puberty scores [26, 27] or SGA did not improve this model. Significance was a two–sided *P* value of ≤0.05.

## Results

### Characteristics of former ELBW children and controls

The cohort of former ELBW children consisted of 140 extreme preterm birth survivors (cases) (Fig 1). Of cases eligible for inclusion in young adolescence, 93 accepted the study invitation. There were minor differences in some characteristics of early life exposures between the children followed (n = 93) *vs*. not followed (n = 47). Analyzed children had a smaller head circumference at birth (23.7 *vs*. 24.4 cm, *P* = 0.012), lower Apgar scores (7.7 *vs*. 8.3, *P* = 0.004) and more ventilation days (19.6 *vs*. 12.6, *P* = 0.046) and oxygen need (51 *vs*. 38%, *P* = 0.041). Maternal antenatal lung maturation (i.e. two doses of intramuscular betamethasone with 24h between both administrations) was less frequent in the analyzed children (79.8 *vs*. 88.9%, *P* = 0.006) as well as for pre–eclampsia (11.1 *vs*. 29.3%, p = 0.018) (S1 Table). Similarly, when compared cases to controls in young adolescence, mean values of age (11.3 *vs*. 10.9 years, *P* = 0.025), height (145.1 *vs*. 149.2 cm, *P* = 0.027), weight (36.7 *vs*. 39.9 kg, *P* = 0.012) and head circumference (51.7 *vs*. 53.4 cm, *P*<0.001) differed significantly (Table 1).

In ELBW cases, neurocognitive performance at the corrected age of 24 months was 93.6 (SD 22.2) and motor performance was 85.1 (SD 22.1). Compared to controls, neurocognitive outcome at 11 years showed matrix reasoning and spatial orientation of 47.5 (SD 6.5) and 45.6 (SD 8.6) in cases *vs*. 53.1 (SD 7.4) and 55.9 (SD 9.4) in controls (both *P*<0.001). The total IQ–equivalent was 92.5 (SD 13.1) in cases *vs*. 108.7 (SD 14.1) in controls (*P*<0.001). Of the cases, n = 10 were following special education, mainly because of lower IQ, whereas all controls were recruited from regular primary education. Paternal educational levels were equally distributed among cases and controls (low 6.8 *vs*. 2.5%; middle 45.2 *vs*. 51.2%; high 47.9 *vs*. 46.3%; *P* = 0.389) as well as maternal educational levels (low 6.7 *vs*. 0.0%; middle 38.7 *vs*. 41.8%; high 54.7 *vs*. 58.2%; *P* = 0.06).

Body composition was different in cases compared to controls in young adolescence: former ELBW children had a higher percentage body fat (but lower total body weight) and lower percentage lean body weight (weight 36.7 *vs*. 39.9 kg, *P* = 0.012; fat mass 24.6 *vs*. 19.2%, *P* = 0.001; lean mass 75.1 *vs*. 80.5%, *P* = 0.001). Moreover, cases had lower grip strength and a more advanced puberty (Table 1). Analyses stratified by sex, generated results (data not shown) consistent with those reported in Table 1.

### Growth patterns in cases

**Fig 2:**
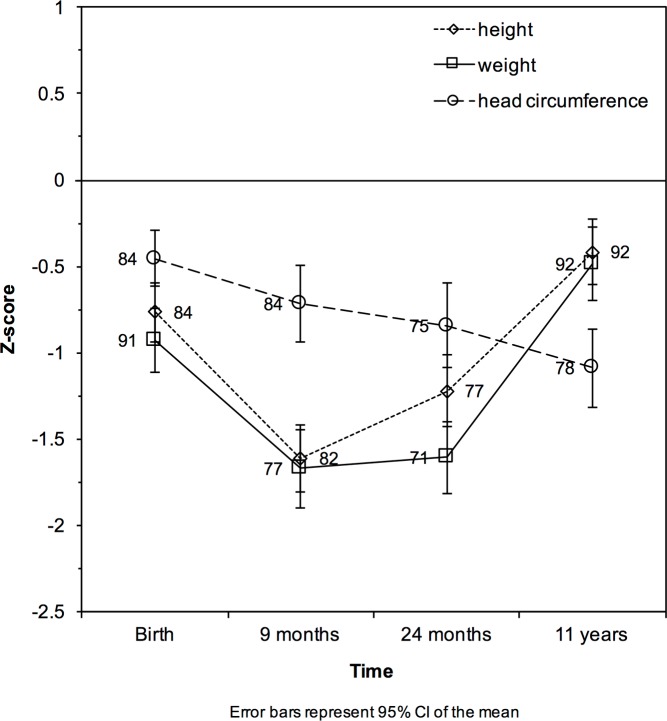
Mean Z–scores of height, weight and head circumference over time).

For cases only, we investigated growth patterns from birth to young adolescence (11 years). ELBW neonates displayed extra–uterine growth restriction with mean Z–scores for height, weight and head circumference of –0.77, –0.93 and –0.46 at birth, –1.61, –1.67 and –0.72 at 9 months, –1.22, –1.61 and –0.84 at 24 months, and –0.42, –0.49 and –1.09 at 11 years (Fig 2). Mean Z–scores for height, weight and head circumference differed significantly between time points (all *P*<0.01), with the exception of Z–scores for height and weight at birth and 11 years and for head circumference between birth and 9 months and between 24 months and 11 years. ELBW cases s displayed extra–uterine growth restriction in height and weight with subsequent catch–up growth between 24 months and 11 years (Fig 2).

**Fig 3:**
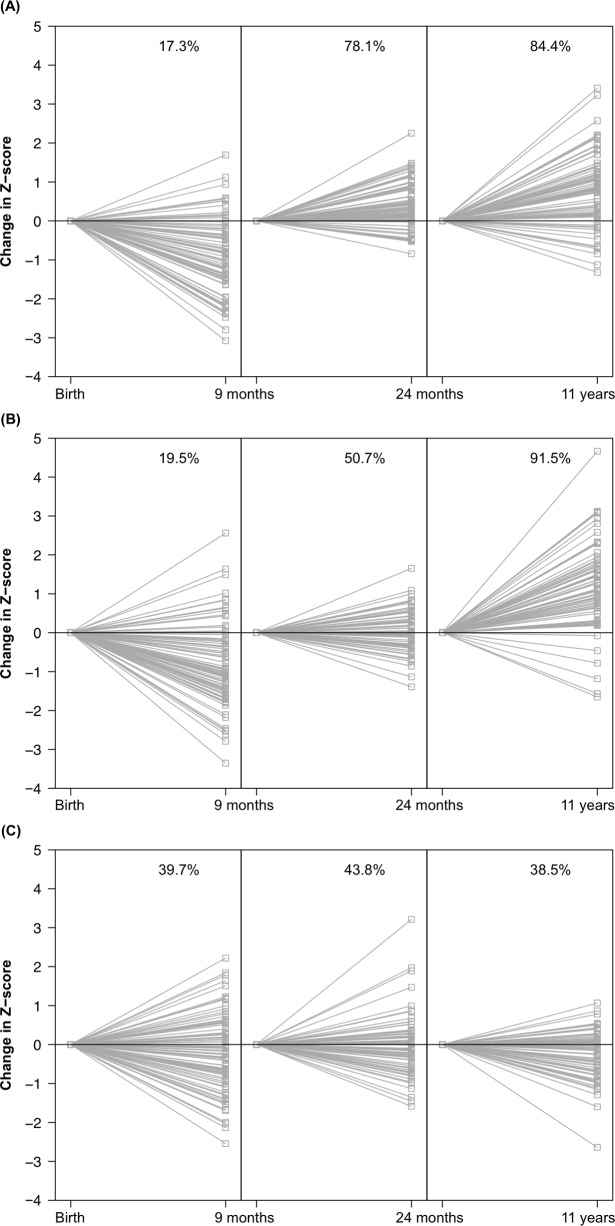
Z–score trends in individual cases from birth till 11 years). (A) Z-scores for height, (B) Z-scores for weight, and (C) Z-scores for head circumference.

Fig 3 showed the individual directions of the change in Z-score. Most cases showed growth failure during the first two years of life and a positive Z–score change from 24 months till 11 years: 91.5% for height and 84.4% for weight, but not for head circumference (38.5%).

Catch–up growth (i.e. ΔZ–score >0.67 SDS) for weight was achieved during the first two years of life in 9.9% of cases, for length in 13.7% of cases and for head circumference in 14.1% of cases. During childhood (2 years– 11 years) another 73.2% of cases showed catch–up growth for weight, 59.7% for length and 4.6% for head circumference.

We performed a subgroup analysis in ELBW cases that were either appropriate for gestational age (AGA) or small for gestational age (SGA). Mean Z–scores for height and weight of ELBW cases AGA or SGA only differed significantly at birth, as per definition, and at 9 months (data not shown). However, analysis of target height (Z-score) at 11 years of AGA and SGA cases at 11 years showed that the height in SGA cases was below target height [current height –0.22 (SD 0.89) *vs*. target height 0.16 in SGA cases (SD 0.77), *P* = 0.038], indicating the inability of SGA cases to (fully) catch–up.

### Catch–up growth associations with neurocognitive outcome and body composition

Analyses relating catch–up growth for weight, height and head circumference to neurocognitive performance did not show significant correlations (data not shown). Notwithstanding this, cases showed lower neurocognitive performance and a higher fat percentage compared to controls.

Catch–up growth for weight and height during the first two years of life was significantly associated with a lower percentage body fat in young adolescence. The percentage body fat in young adolescence was 16.8% if catch-up growth for weight occurred during the first two years of life versus 25.7% if there was no catch-up growth for weight during the first two years of life (*P*<0.001). The percentage body fat was also associated with catch-up growth for height (20.9% catch-up *vs*. 25.8% no catch-up, *P* = 0.049) (Table 2). Catch–up growth during childhood (2–11 years) was associated with the percentage body fat in young adolescence for weight only and the difference was less pronounced (23.7% catch-up for weight *vs*. 29.4%, no catch-up for weight, *P =* 0.036). We observed that catch-up growth for head circumference during the first 9 months of life was associated with a lower body fat percentage as well.

**Table 2:** Difference in catch-up growth and no catch-up growth during childhood versus body fat in young adolescence in former ELBW children.

	Catch-up growth^‡^			No catch-up growth			
	*n*	*mean*^*§*^	*95% CI*	*n*	*mean*^*§*^	*95% CI*	*P* ^*¶*^
**Weight between**							
0–9 months	6	18.7	14.0–23.4	62	25.3	22.9–27.7	0.012
0–24 months	6	16.8	13.9–19.8	58	25.7	23.4–28.0	<0.001
2–11 years	51	23.7	21.3–26.1	13	29.4	23.9–25.0	0.036
**Height between**							
0–9 months	2	19.4	n/a	67	25.5	23.2–27.8	0.38
0–24 months	9	20.9	16.4–25.4	57	25.8	23.3–28.3	0.049
2–11 years	42	23.9	21.0–26.7	26	26.7	23.1–30.3	0.21
**Head circumference between**							
0–9 months	13	20.1	15.9–24.3	57	26.6	24.1–29.2	0.025
0–24 months	10	22.0	15.7–28.4	55	26.3	23.7–28.8	0.19
2–11 years	2	28.2	n/a	57	25.7	23.2–28.2	0.09

^‡^Catch-up growth: change in Z-score between time points >0.67

^§^Percentage body fat at 11 years controlled for height, weight, sex, age, and height × weight

^¶^Student’s t-test (two-tailed)

## Discussion

In the current study of ELBW infants, we reported on height, weight and head circumference at birth and growth patterns of these anthropometric measurements at 9 and 24 months and at 11 years of age. At the latest follow–up point, we compared achieved growth, body composition and neurocognition between ELBW children and normal birth weight controls of equal sex and age and explored association between early catch–up (first two years of life) and outcome at 11 years. The findings of the current study confirmed that the former ELBW children: (i) had difficulties in catching up growth; (ii) showed lower neurocognitive performance in childhood and young adolescence; and (iii) at 11 years, compared to normal birth weight controls, showed lower grip strength and percentage lean body weight, advanced puberty scores, but a higher percentage body fat, a potential risk factor for adverse health outcomes in adulthood. Our key finding was that catch–up growth for weight and height in ELBW children in the first two years of life is associated with a lower percentage body fat and is therefore likely to be beneficial.

Former ELBW children had difficulties to catch–up. Catch–up growth can be defined in terms of body height and weight (in centimeters or kilograms). However, using Z–scores comparing an individual to sex–and age–specific distributions in the general population is a more generally accepted approach [6, 30]. According to the available literature, growth patterns in children born preterm showed initial growth failure [31] followed by increased growth velocity during the first years of life [32]. However, catch–up remains usually incomplete, especially in children born small for gestational age (SGA). Our study moved beyond currently available data by showing growth restriction in former ELBW children, irrespective of SGA or appropriate for gestational age (AGA) status at birth, with catch–up growth after 24 months. At 11 years, body height of ELBW children was still below the population average as exemplified by a negative Z–score (Fig 2). In particular, children born SGA still had a body height significantly below target. The qualification SGA is commonly applied as a proxy for intra–uterine growth restriction. Our primary analyses comparing growth patterns of SGA and AGA ELBW children did not reveal any significant differences (data not shown). However, if we used target height as computed from the parents’ body height, SGA children failed to reach this target height, similar to cohorts with birth weight below 1500 grams [33, 34].

The presence of catch–up growth (weight, height or head circumference) was not associated with differences in neurocognitive outcome in ELBW cases. In our cohort, the extent of extra–uterine growth restriction for the head circumference was more blunted compared to height and weight (Fig 2). However, this ‘preserved’ head circumference did not (fully) preserve neurocognitive development. We could not confirm the finding that catch–up growth was associated with worse neurocognitive outcome [35, 36]. There was no difference in IQ equivalent between ELBW children with and without catch–up growth at 11 years (data not shown). One possible explanation is that many (environmental) confounders for neurocognitive development in childhood are at play and none can be assumed equal among the included cases to justify such testing. Another explanation is that too many children still fail to catch–up (23–95% for weight, height, head circumference in early childhood or adolescence, Fig 2) to show relevant differences. It is still unclear which period (pre–, peri–and/or postnatal) will have the most relevant implications for later neurocognitive outcome (reviewed in [8]). However, neurocognitive impairment after preterm birth remains a matter of concern [37]. Unfortunately, and despite major improvements in neonatal intensive care survival, we could not find full catch–up growth and preserved neurocognitive outcome in this cohort of ELBW survivors born between 2000–2005.

At 11 years, grip strength was lower in former ELBW children compared to controls with normal birth weight. Motor assessment Bayley scores at 24 months (for cases) provided more comprehensive information than the single grip strength test at 11 years. Test results at 24 months can therefore not be extrapolated as representative of motor development approximately one decade later. However, at 11 years, lower grip strength in childhood may result from a decreased exercise capacity in former ELBW children [38, 39]. Clemm [38] and Welsh and coauthors [39] both showed lower oxygen consumption as measured by spirometry. The first study showed lower exercise capacity in extreme preterm children as well [38]. The lower grip strength might be a reflection of the long–term pathophysiological consequences, including the lower muscle mass in former preterms. Moreover, Fricke et al. showed an association between anthropometrics and grip strength at 7 years in very low birth weight (<1500 grams) prematurely born infants [40]. Similarly, grip force and peak jump power were significantly lower compared to the reference population in their study [38].

Our current study also confirmed a more advanced puberty in former ELBW children, compared to their age–matched controls. Ibánez *et al*. showed a more advanced pubertal development of 5–10 months in SGA girls [41]. Pubertal development and especially the impact of preterm birth on puberty are difficult to study, since the influences are multifactorial with interactions between nature and nurture [42]. However, early weight gain in infancy might start a cascade of disrupted pubertal development leading to an adipose body composition and decreased fertility even in the absence of obesity [43].

Catch–up growth for weight and height was associated with a lower percentage body fat during the first two years of life. As index of growth, not only body height and weight, but also body composition should be considered, because catch–up growth and fat mass accretion do not occur simultaneously [11]. In preterm children, early weight–gain during the first three months of life and from three months till one year was predictive for body mass index in adulthood, independently of height [11]. Preterm neonates assessed at birth have an increased total body adiposity compared to term infants [44]. We replicated this observation in young adolescence (Table 1). Despite the lower body mass index in former ELBW children (but also lower height), our extensive body composition measurements at 11 years of age confirmed a higher percentage body fat in childhood. Catch–up growth for weight and height was associated with a lower percentage body fat. As suggested by Breij *et al*. [45], there might be a critical window for adiposity development in the first three months of life. We may extrapolate these results to preterm children. During this window, preterm children are *ex utero* and due to this too early interruption of normal *in utero* development, they might accumulate fat mass at the detriment of lean body mass (i.e. muscle mass). This might be reflected in both a decreased exercise capacity (multifactorial, reviewed in [46]) and an increased cardiovascular risk profile (increased fat mass, reviewed in [14]). A possible mechanism in this pathophysiological process is the (impaired) microcirculation. The microcirculation is the primary interface between the circulating blood and capillary density is lower in several organs (f.e. the kidneys, the brain, the lungs, the retina) in preterm children [47, 48]. This puts these children at risk for cardiovascular events later in life [5], possibly through epigenetic changes [49].

A strong point of the current study was that the ELBW children were followed–up by the same team, using validated and standardized instruments from birth until approximately 11 years and that at 11 years their growth pattern could be compared not only with the population average, but also with controls. However, our study must also be interpreted within the context of its potential limitations. First, at 11 years we did not extensively assess motor development. We only assessed one aspect (i.c. grip strength) of motor development. However, in adolescents with cystic fibrosis, grip strength performs well as index of motor strength, correlating with other indexes, such as lung capacity parameters and fitness [50]. Second, children in the control group were slightly younger (10.9 *vs*. 11.3 years). However, after correction for age, the results remained significant (*P* = 0.025, Table 1). Moreover, a 4–month period is unlikely to offer the whole explanation, in particular in view of the literature summarized above. Third, the Bayley Scales of Infant Development and the grip strength test measure different aspects of motor performance [40]. However, both tests are appropriate for the age of the children at which they were administered. A limitation of our study is that we could not assess longitudinal changes in ELBW infants because differences in the test design and not in controls, because no test was administered at two years of age. Fourth, we could not fully exclude a selection bias in the recruitment. Analyzed children had a smaller head circumference, lower Apgar scores and more ventilation days and oxygen need. Maternal antenatal lung maturation was less frequent in the analyzed children as well as pre–eclampsia. However, since differences are in both directions (for example more ventilation days but less pre–eclampsia in the analyzed children), we assume that selection was at random. Being aware of these limitations, we still think our observations are relevant for the follow–up of former extremely low birth weight infants.

For the follow–up of these ELBW adolescents and future studies, as stated in literature, being short or becoming short during the first two years of life increases the risk of adult short stature [51]. Since best results of growth hormone therapy are obtained before the onset of puberty [52], parents and caregivers should be aware of a possible earlier onset of puberty in former ELBW adolescents. In addition, higher weight at birth (i.e. being non–small–for–gestational–age) might be the key to a higher percentage lean body weight as a model of a healthy start of life [53] in contrast with children SGA who need to catch–up [54]. Insulin sensitization in children with early neonatal weight gain might be the key to reduce the cardiovascular risk profile later in life [43]. Further studies should elucidate this insulin sensitization in primary prevention of cardiovascular events in children born preterm.

## Conclusions

The current study of ELBW infants reported on height, weight and head circumference at birth and described the growth patterns of these anthropometric measurements at 9 and 24 months and at 11 years of age. At the lastest follow–up point, compared to normal birth weight controls, former ELBW children still have height, weight and head circumference below target, show lower grip strength, lower neurocognitive performance, but display more advanced puberty. ELBW adolescents showed a higher percentage body fat, a potential risk factor for adverse health outcomes in adulthood. Our key finding was that catch–up growth for weight in ELBW children in the first two years of life was associated with a lower percentage body fat and is therefore likely to be beneficial.

## Supporting information

S1 Table Differences in characteristics of recruited/not recruited of the initial cohort of Extremely Low Birth Weight (ELBW) survivors. (DOCX)

## References

[1] Feigin VL, Roth GA, Naghavi M, Parmar P, Krishnamurthi R, Chugh S, et al Global burden of stroke and risk factors in 188 countries, during 1990–2013: a systematic analysis for the Global Burden of Disease Study 2013. The Lancet Neurology. 2016. 10.1016/S1474-4422(16)30073-4 27291521

[2] McGorrian C, Yusuf S, Islam S, Jung H, Rangarajan S, Avezum A, et al Estimating modifiable coronary heart disease risk in multiple regions of the world: the INTERHEART Modifiable Risk Score. European heart journal. 2011;32(5):581–9. 10.1093/eurheartj/ehq448 21177699

[3] Yusuf S, Hawken S, Ounpuu S, Bautista L, Franzosi MG, Commerford P, et al Obesity and the risk of myocardial infarction in 27,000 participants from 52 countries: a case-control study. Lancet (London, England). 2005;366(9497):1640–9. 10.1016/S0140-6736(05)67663-5 16271645

[4] Sniderman AD, Islam S, McQueen M, Pencina M, Furberg CD, Thanassoulis G, et al Age and Cardiovascular Risk Attributable to Apolipoprotein B, Low-Density Lipoprotein Cholesterol or Non-High-Density Lipoprotein Cholesterol. Journal of the American Heart Association. 2016;5(10). 10.1161/JAHA.116.003665 PMC5121475 27737874

[5] Ligi I, Grandvuillemin I, Andres V, Dignat-George F, Simeoni U. Low birth weight infants and the developmental programming of hypertension: a focus on vascular factors. Seminars in perinatology. 2010;34(3):188–92. 10.1053/j.semperi.2010.02.002 20494734

[6] Ong KK, Kennedy K, Castaneda-Gutierrez E, Forsyth S, Godfrey KM, Koletzko B, et al Postnatal growth in preterm infants and later health outcomes: a systematic review. Acta paediatrica (Oslo, Norway: 1992). 2015;104(10):974–86. 10.1111/apa.13128 PMC5054880 26179961

[7] Lapillonne A, Griffin IJ. Feeding preterm infants today for later metabolic and cardiovascular outcomes. The Journal of pediatrics. 2013;162(3 Suppl):S7–16. 10.1016/j.jpeds.2012.11.048 23445851

[8] Ranke MB, Krageloh-Mann I, Vollmer B. Growth, head growth, and neurocognitive outcome in children born very preterm: methodological aspects and selected results. Developmental medicine and child neurology. 2015;57(1):23–8. 10.1111/dmcn.12582 25251724

[9] Yamakawa T, Itabashi K, Kusuda S. Mortality and morbidity risks vary with birth weight standard deviation score in growth restricted extremely preterm infants. Early human development. 2015;92:7–11. 10.1016/j.earlhumdev.2015.10.019 26615548

[10] Embleton ND, Korada M, Wood CL, Pearce MS, Swamy R, Cheetham TD. Catch-up growth and metabolic outcomes in adolescents born preterm. Archives of disease in childhood. 2016. 10.1136/archdischild-2015-310190 27288431

[11] Euser AM, Finken MJ, Keijzer-Veen MG, Hille ET, Wit JM, Dekker FW. Associations between prenatal and infancy weight gain and BMI, fat mass, and fat distribution in young adulthood: a prospective cohort study in males and females born very preterm. The American journal of clinical nutrition. 2005;81(2):480–7. 15699238 10.1093/ajcn.81.2.480

[12] Gianni ML, Roggero P, Liotto N, Taroni F, Polimeni A, Morlacchi L, et al Body composition in late preterm infants according to percentile at birth. Pediatric research. 2016;79(5):710–5. 10.1038/pr.2015.273 26717003

[13] Euser AM, de Wit CC, Finken MJ, Rijken M, Wit JM. Growth of preterm born children. Hormone research. 2008;70(6):319–28. 10.1159/000161862 18953169

[14] Kerkhof GF, Hokken-Koelega ACS. Rate of neonatal weight gain and effects on adult metabolic health. Nat Rev Endocrinol. 2012;8(11):689–92. 10.1038/nrendo.2012.168 22987159

[15] Devlieger H, De Pourcq L, Casneuf A, Vanhole C, de Zegher F, Jaeken J, et al Standard two-compartment formulation for total parenteral nutrition in the neonatal intensive care unit: A fluid tolerance based system. Clinical nutrition (Edinburgh, Scotland). 1993;12(5):282–6. 10.1016/0261-5614(93)90047-8 16843327

[16] Agostoni C, Buonocore G, Carnielli VP, De Curtis M, Darmaun D, Decsi T, et al Enteral nutrient supply for preterm infants: commentary from the European Society of Paediatric Gastroenterology, Hepatology and Nutrition Committee on Nutrition. Journal of pediatric gastroenterology and nutrition. 2010;50(1):85–91. 10.1097/MPG.0b013e3181adaee0 19881390

[17] George I, Mekahli D, Rayyan M, Levtchenko E, Allegaert K. Postnatal trends in creatinemia and its covariates in extremely low birth weight (ELBW) neonates. Pediatric nephrology (Berlin, Germany). 2011;26(10):1843–9. 10.1007/s00467-011-1883-0 21499946

[18] Raaijmakers A, Petit T, Gu Y, Zhang Z, Wei F, Cools B, et al Design and feasibility of 'PREMATurity as predictor of children's Cardiovascular-renal Health' (PREMATCH): A pilot study. Blood pressure. 2015;24(5):275–83. 10.3109/08037051.2015.1053220 26107770 PMC4673568

[19] Van der Meulen B, Ruiter S, Lutje Spelberg H, Smrkovsky M. BSID-II-NL, Dutch Manual. Lisse: Swets 2002.

[20] Wechsler DNJA. WNV NL. Wechsler Nonverbal Scale of ability. Nederlandstalige bewerking. Technische handleiding. Amsterdam: Pearson; 2008.

[21] Roelants M, Hauspie R, Hoppenbrouwers K. References for growth and pubertal development from birth to 21 years in Flanders, Belgium. Annals of human biology. 2009;36(6):680–94. 10.3109/03014460903049074 19919503

[22] Tanner JM, Goldstein H, Whitehouse RH. Standards for Children's Height at Age 2 to 9 years allowing for height of Parents. Archives of disease in childhood. 1970;45(244):819. 10.1136/adc.45.244.819-b PMC1647447 21032457

[23] Ong KK, Ahmed ML, Emmett PM, Preece MA, Dunger DB. Association between postnatal catch-up growth and obesity in childhood: prospective cohort study. BMJ (Clinical research ed). 2000;320(7240):967–71. 10.1136/bmj.320.7240.967 PMC27335 10753147

[24] Segal KR, Burastero S, Chun A, Coronel P, Pierson RN Jr., Wang J. Estimation of extracellular and total body water by multiple-frequency bioelectrical-impedance measurement. The American journal of clinical nutrition. 1991;54(1):26–9. 2058583 10.1093/ajcn/54.1.26

[25] Stewart SP, Bramley PN, Heighton R, Green JH, Horsman A, Losowsky MS, et al Estimation of body composition from bioelectrical impedance of body segments: comparison with dual-energy X-ray absorptiometry. The British journal of nutrition. 1993;69(3):645–55. 8329341 10.1079/bjn19930066

[26] Marshall WA, Tanner JM. Variations in pattern of pubertal changes in girls. Archives of disease in childhood. 1969;44(235):291–303. 5785179 10.1136/adc.44.235.291 PMC2020314

[27] Marshall WA, Tanner JM. Variations in the pattern of pubertal changes in boys. Archives of disease in childhood. 1970;45(239):13–23. 5440182 10.1136/adc.45.239.13 PMC2020414

[28] Hoaglin DC, Iglewicz B, Tukey JW. Performance of Some Resistant Rules for Outlier Labeling. Journal of the American Statistical Association. 1986;81(396):991–9.

[29] Hoaglin DC, Iglewicz B. Fine-Tuning Some Resistant Rules for Outlier Labeling. Journal of the American Statistical Association. 1987;82(400):1147–9.

[30] Wit JM, Boersma B. Catch-up growth: definition, mechanisms, and models. Journal of pediatric endocrinology & metabolism: JPEM. 2002;15 Suppl 5:1229–41. 12510974

[31] Cole TJ, Statnikov Y, Santhakumaran S, Pan H, Modi N. Birth weight and longitudinal growth in infants born below 32 weeks' gestation: a UK population study. Archives of disease in childhood Fetal and neonatal edition. 2014;99(1):F34–40. 10.1136/archdischild-2012-303536 23934365 PMC3888637

[32] Hokken-Koelega AC, De Ridder MA, Lemmen RJ, Den Hartog H, De Muinck Keizer-Schrama SM, Drop SL. Children born small for gestational age: do they catch up? Pediatric research. 1995;38(2):267–71. 10.1203/00006450-199508000-00022 7478827

[33] Guellec I, Marret S, Baud O, Cambonie G, Lapillonne A, Roze JC, et al Intrauterine Growth Restriction, Head Size at Birth, and Outcome in Very Preterm Infants. The Journal of pediatrics. 2015;167(5):975–81.e2. 10.1016/j.jpeds.2015.08.025 26384436

[34] Vasquez-Garibay EM, Larios Del Toro YE, Larrosa-Haro A, Troyo-Sanroman R. Anthropometric indicators of nutritional status and growth in very low birth-weight premature infants hospitalized in a neonatal intensive care unit. Nutricion hospitalaria. 2014;30(2):410–6. 10.3305/nh.2014.30.2.7373 25208797

[35] Marlow N, Wolke D, Bracewell MA, Samara M. Neurologic and developmental disability at six years of age after extremely preterm birth. The New England journal of medicine. 2005;352(1):9–19. 10.1056/NEJMoa041367 15635108

[36] Wood NS, Marlow N, Costeloe K, Gibson AT, Wilkinson AR. Neurologic and developmental disability after extremely preterm birth. EPICure Study Group. The New England journal of medicine. 2000;343(6):378–84. 10.1056/NEJM200008103430601 10933736

[37] Jarjour IT. Neurodevelopmental outcome after extreme prematurity: a review of the literature. Pediatric neurology. 2015;52(2):143–52. 10.1016/j.pediatrneurol.2014.10.027 25497122

[38] Clemm HH, Vollsaeter M, Roksund OD, Markestad T, Halvorsen T. Adolescents who were born extremely preterm demonstrate modest decreases in exercise capacity. Acta paediatrica (Oslo, Norway: 1992). 2015;104(11):1174–81. 10.1111/apa.13080 26096772

[39] Welsh L, Kirkby J, Lum S, Odendaal D, Marlow N, Derrick G, et al The EPICure study: maximal exercise and physical activity in school children born extremely preterm. Thorax. 2010;65(2):165–72. 10.1136/thx.2008.107474 19996340

[40] Fricke O, Roedder D, Kribs A, Tutlewski B, von Kleist-Retzow JC, Herkenrath P, et al Relationship of muscle function to auxology in preterm born children at the age of seven years. Hormone research in paediatrics. 2010;73(5):390–7. 10.1159/000308173 20389111

[41] Ibanez L, de Zegher F. Puberty and prenatal growth. Molecular and cellular endocrinology. 2006;254–255:22–5. 10.1016/j.mce.2006.04.010 16757105

[42] Paul A, Deans R, Viner R, Creighton SM. Pubertal development and sexuality in female adolescents born preterm: a review of the literature. International journal of adolescent medicine and health. 2011;23(3):175–9. 22191180 10.1515/ijamh.2011.040

[43] de Zegher F, Ibanez L. Prenatal growth restraint followed by catch-up of weight: a hyperinsulinemic pathway to polycystic ovary syndrome. Fertility and sterility. 2006;86 Suppl 1:S4–5. 16798286 10.1016/j.fertnstert.2006.03.013

[44] Uthaya S, Thomas EL, Hamilton G, Dore CJ, Bell J, Modi N. Altered adiposity after extremely preterm birth. Pediatric research. 2005;57(2):211–5. 10.1203/01.PDR.0000148284.58934.1C 15611357

[45] Breij LM, Kerkhof GF, De Lucia Rolfe E, Ong KK, Abrahamse-Berkeveld M, Acton D, et al Longitudinal fat mass and visceral fat during the first 6 months after birth in healthy infants: support for a critical window for adiposity in early life. Pediatric obesity. 2016. 10.1111/ijpo.12139 PMC6186414 27072083

[46] Lowe J, Cousins M, Kotecha SJ, Kotecha S. Physical activity outcomes following preterm birth. Paediatric respiratory reviews. 2016. 10.1016/j.prrv.2016.08.012 27746158

[47] Bonamy AK, Martin H, Jorneskog G, Norman M. Lower skin capillary density, normal endothelial function and higher blood pressure in children born preterm. Journal of internal medicine. 2007;262(6):635–42. 10.1111/j.1365-2796.2007.01868.x 17986202

[48] Lewandowski AJ, Davis EF, Yu G, Digby JE, Boardman H, Whitworth P, et al Elevated blood pressure in preterm-born offspring associates with a distinct antiangiogenic state and microvascular abnormalities in adult life. Hypertension. 2015;65(3):607–14. 10.1161/HYPERTENSIONAHA.114.04662 25534704

[49] Simeoni U, Ligi I, Buffat C, Boubred F. Adverse consequences of accelerated neonatal growth: cardiovascular and renal issues. Pediatric nephrology (Berlin, Germany). 2011;26(4):493–508. 10.1007/s00467-010-1648-1 20938692

[50] Wells GD, Wilkes DL, Schneiderman JE, Thompson S, Coates AL, Ratjen F. Physiological correlates of pulmonary function in children with cystic fibrosis. Pediatric pulmonology. 2014;49(9):878–84. 10.1002/ppul.22928 24166871

[51] Luo ZC, Albertsson-Wikland K, Karlberg J. Length and body mass index at birth and target height influences on patterns of postnatal growth in children born small for gestational age. Pediatrics. 1998;102(6):E72 9832600 10.1542/peds.102.6.e72

[52] Simon D, Leger J, Carel JC. Optimal use of growth hormone therapy for maximizing adult height in children born small for gestational age. Best practice & research Clinical endocrinology & metabolism. 2008;22(3):525–37. 18538291 10.1016/j.beem.2008.03.003

[53] de Zegher F, Diaz M, Lopez-Bermejo A, Ibanez L. Recognition of a sequence: more growth before birth, longer telomeres at birth, more lean mass after birth. Pediatric obesity. 2016. 10.1111/ijpo.12137 27071945

[54] Ibanez L, Lopez-Bermejo A, Diaz M, de Zegher F. Catch-up growth in girls born small for gestational age precedes childhood progression to high adiposity. Fertility and sterility. 2011;96(1):220–3. 10.1016/j.fertnstert.2011.03.107 21549368

