# Correction: Catch–up growth in the first two years of life in Extremely Low Birth Weight (ELBW) infants is associated with lower body fat in young adolescence

**DOI:** 10.1371/journal.pone.0196441

**Published:** 2018-04-19

**Authors:** Anke Raaijmakers, Lotte Jacobs, Maissa Rayyan, Theun Pieter van Tienoven, Els Ortibus, Elena Levtchenko, Jan A. Staessen, Karel Allegaert

In S1 Table, the columns listing the differences in the characteristics of the recruited and non-recruited initial cohort of Extremely Low Birth Weight (ELBW) survivors are incorrectly switched between groups. As a result, the absolute numbers given in the second part of the table are incorrectly derived by multiplying the percentage of occurrence of a characteristic with the wrong group size. Please see the corrected S1 Table here.

In light of this error, several sentences in the body of the paper should be corrected.

In the Characteristics of former ELBW children and controls subsection of the Results section, the fourth and fifth sentences should read: Analyzed children had a larger head circumference at birth (24.4 *vs*. 23.7 cm, *P* = 0.012), higher Apgar scores (8.3 *vs*. 7.7, *P* = 0.004) and less ventilation days (12.6 *vs*. 19.6, *P* = 0.046) and oxygen need (38 *vs*. 51%, *P* = 0.041). Maternal antenatal lung maturation (i.e. two doses of intramuscular betamethasone with 24h between both administrations) was more frequent in the analyzed children (88.9 *vs*. 79.8%, *P* = 0.006) as well as for pre–eclampsia (29.3 *vs*. 11.1%, p = 0.018) (S1 Table).

In the seventh paragraph of the Discussion section, the thirteenth, fourteenth, and fifteenth sentences should read: Analyzed children had a larger head circumference, higher Apgar scores and less ventilation days and oxygen need. Maternal antenatal lung maturation was more frequent in the analyzed children as well as pre–eclampsia. However, since differences are in both directions (for example less ventilation days but more pre–eclampsia in the analyzed children), we assume that selection was at random.

## Supporting information

S1 Table Differences in characteristics of recruited/not recruited of the initial cohort of Extremely Low Birth Weight (ELBW) survivors. (DOCX)
