# Supplementary material for: Catch–up growth in the first two years of life in Extremely Low Birth Weight (ELBW) infants is associated with lower body fat in young adolescence
Source: PLoS One. 2017 Mar 9;12(3):e0173349. doi: 10.1371/journal.pone.0173349 (PMC5344416; doi:10.1371/journal.pone.0173349)
Supplement: S1 Table — (DOCX) [file pone.0173349.s001.docx]

**Supplement**

**S1 Table** Differences in characteristics of recruited/not recruited of the initial cohort of Extremely Low Birth Weight (ELBW) survivors

|  | **ELBW cohort survivors (n=140)** | | |
| --- | --- | --- | --- |
|  | Analyzed (n=93) | Not analyzed (n=47) | *P* |
| **Mean±SD of characteristic** |  |  |  |
| Gestational age (weeks) | 27.0±2.2 | 27.4±1.9 | 0.39 |
| Birth weight (g) | 780.9±151.1 | 796.2±138.1 | 0.55 |
| Birth length (cm) | 33.3±2.5 | 33.5±2.5 | 0.61 |
| Birth head circumference (cm) | 23.7±1.4 | 24.4±1.5 | **0.012** |
| Apgar score 1 min | 5.1±2.4 | 6.1±2.2 | **0.024** |
| Apgar score 5 min | 7.7±1.4 | 8.3±0.9 | **0.004** |
| Apgar score 10 min | 8.8±0.7 | 9.0±0.8 | 0.15 |
| Any oxygen need (days) | 51.0±39.1 | 38.2±34.3 | **0.041** |
| Ventilation days | 19.6±22.2 | 12.6±17.8 | **0.046** |
| Days to full enteral feeding | 43.2±25.1 | 40.2±23.9 | 0.42 |
| **Number with characteristic, n (%)** |  |  |  |
| Tocolysis | 28 (30.2) | 15 (32.6) | 0.79 |
| Antenatal lung maturation^a^ | 74 (79.8) | 42 (88.9) | **0.006** |
| Pre–eclampsia | 10 (11.1) | 14 (29.3) | **0.018** |
| Premature rupture of membranes | 22 (23.3) | 9 (19.8) | 0.64 |
| Chorioamnionitis | 12 (13.3) | 2 (4.4) | 0.06 |
| Postnatal steroids | 58 (62.2) | 24 (51.1) | 0.22 |
| Ibuprofen use | 49 (52.2) | 23 (49.5) | 0.76 |
| Any intraventricular hemorrhage | 22 (23.9) | 11 (22.8) | 0.89 |
| Retinopathy of prematurity | 29 (31.1) | 9 (18.7) | 0.10 |
| Retinopathy of prematurity (≥stage 3) | 23 (25.0) | 9 (18.7) | 0.40 |
| Small for gestational age | 24 (26.1) | 18 (39.1) | 0.13 |
| Intubation at birth | 64 (68.9) | 31 (65.2) | 0.67 |

^a^Antenatal lung maturation was two days of intramuscular betamethasone (reference 15). Extremely Low Birth Weight, ELBW. *P* values are given for the comparison between ELBW cases and controls (T–test or Mann–Whitney–U test for continuous variables and Pearson Chi square test for categorical variables).
