# Supplementary material for: Correction: Catch–up growth in the first two years of life in Extremely Low Birth Weight (ELBW) infants is associated with lower body fat in young adolescence
Source: PLoS One. 2018 Apr 19;13(4):e0196441. doi: 10.1371/journal.pone.0196441 (PMC5908076; doi:10.1371/journal.pone.0196441)
Supplement: S1 Table — (DOCX) [file pone.0196441.s001.docx]

**Supplement**

**S1 Table** Differences in characteristics of recruited/not recruited of the initial cohort of Extremely Low Birth Weight (ELBW) survivors

|  | **ELBW cohort survivors (n=140)** | | |
| --- | --- | --- | --- |
|  | Analyzed (n=93) | Not analyzed (n=47) | *P* |
| **Mean±SD of characteristic** |  |  |  |
| Gestational age (weeks) | 27.4±1.9 | 27.0±2.2 | 0.39 |
| Birth weight (g) | 796.2±138.1 | 780.9±151.1 | 0.55 |
| Birth length (cm) | 33.5±2.5 | 33.3±2.5 | 0.61 |
| Birth head circumference (cm) | 24.4±1.5 | 23.7±1.4 | **0.012** |
| Apgar score 1 min | 6.1±2.2 | 5.1±2.4 | **0.024** |
| Apgar score 5 min | 8.3±0.9 | 7.7±1.4 | **0.004** |
| Apgar score 10 min | 9.0±0.8 | 8.8±0.7 | 0.15 |
| Any oxygen need (days) | 38.2±34.3 | 51.0±39.1 | **0.041** |
| Ventilation days | 12.6±17.8 | 19.6±22.2 | **0.046** |
| Days to full enteral feeding | 40.2±23.9 | 43.2±25.1 | 0.42 |
| **Percentage with characteristic (%)** |  |  |  |
| Tocolysis | 32.6 | 30.2 | 0.79 |
| Antenatal lung maturation^a^ | 88.9 | 79.8 | **0.006** |
| Pre–eclampsia | 29.3 | 11.1 | **0.018** |
| Premature rupture of membranes | 19.8 | 23.3 | 0.64 |
| Chorioamnionitis | 4.4 | 13.3 | 0.06 |
| Postnatal steroids | 51.1 | 62.2 | 0.22 |
| Ibuprofen use | 49.5 | 52.2 | 0.76 |
| Any intraventricular hemorrhage | 22.8 | 23.9 | 0.89 |
| Retinopathy of prematurity | 18.7 | 31.1 | 0.10 |
| Retinopathy of prematurity (≥stage 3) | 18.7 | 25.0 | 0.40 |
| Small for gestational age | 39.1 | 26.1 | 0.13 |
| Intubation at birth | 65.2 | 68.9 | 0.67 |

^a^Antenatal lung maturation was two days of intramuscular betamethasone (reference 15). Extremely Low Birth Weight, ELBW. *P* values are given for the comparison between ELBW cases and controls (T–test or Mann–Whitney–U test for continuous variables and Pearson Chi square test for categorical variables).
